# Breast Cancer Knowledge, Attitudes and Practices amongst Women in Qatar

**DOI:** 10.3390/ijerph19073995

**Published:** 2022-03-28

**Authors:** Ehab Hamed, Bayan Alemrayat, Mohamed Ahmed Syed, Suhad Daher-Nashif, Hadi Mohamad Abu Rasheed, Tanya Kane

**Affiliations:** 1 Primary Health Care Corporation, Doha 26555, Qatar; eshamed@phcc.gov.qa (E.H.); bmarayat@phcc.gov.qa (B.A.); masyed@phcc.gov.qa (M.A.S.); 2 Department of Population Medicine, College of Medicine, QU Health, Qatar University, Doha 2713, Qatar; snashif@qu.edu.qa; 3 Qatar Cancer Society, Doha 22944, Qatar; hadi@qcs.qa

**Keywords:** breast cancer screening, breast cancer knowledge and awareness, cancer and oncology, Qatar, Middle East

## Abstract

This cross-sectional study examines knowledge, attitudes, and practices surrounding breast cancer awareness and screening among women residents in Qatar. Females, >18 years old, registered with the Primary Health Care Corporation were invited to complete an Arabic or English online survey using a modified version of the Breast Cancer Awareness Module. Of the 9008 participants, 69% report awareness of breast cancer warning signs, but the results did not substantiate these claims. There remains a disconnect between participants’ perceived awareness of their ability to detect breast cancer and their actual recognition of individual signs and symptoms. Nearly half (45.4%) report rarely or never checking their breasts for abnormalities (44.6%). Breast self-examination (BSE) and Breast Cancer Screening (BCS) uptake is low and many are unaware of the starting age for invitation to Qatar’s BCS program. While only 18% of women report receiving an invitation, 94% attended, indicating that the BCS invitation is a remarkably effective means of improving screening uptake. Policymakers should capitalize on early recognition, which is possible in the youthful population. Broadening awareness campaigns and interventions targeting a broader audience including males, community and religious leaders and healthcare professionals may prove more effective in Arab communities.

## 1. Introduction

Breast cancer is a life-threatening disease affecting women across the globe [1]. It is the most prevalent type of cancer among women and is the primary cause of cancer-related mortality [2]. According to the WHO, 2.3 million women were diagnosed with breast cancer and 685,000 deaths were attributed to it globally in 2020 [3]. Although the disease prevalence is higher in developed countries, three-quarters of the worldwide disease-associated deaths are arising from developing countries, crossing cultures and countries [4,5].

In the Middle East, breast cancer is the most prevalent cancer among females [6]. In Qatar, breast cancer continues to be the most commonly diagnosed cancer among women [7]. According to the estimation of the Global Cancer Observatory, breast cancer ranks highest, accounting for 14.7% of new cancer cases and 37.5% of new cancer cases in females recorded in Qatar during 2020 [7]. Breast cancer is also associated with the highest mortality rates, when compared to all other types, representing 19% of all cancer deaths [7]. Bener et al. report there was a 3.8% rise in new breast cancer cases between the early 1990s to 2006 [8]. Qatar ranks fifth in terms of breast cancer prevalence in the Arab region [9]. These findings demonstrate the need to improve breast cancer awareness and underscores the need to further educate the public about the signs and symptoms, risk factors, prevention, and the importance of screening for early detection.

Early detection of breast cancer is associated with decreased age-adjusted mortality rates of the disease [10]. Women who undergo screening tests potentially discover malignancies at an early stage, facilitating early treatment and ultimately improving survival rates [11]. Similarly, knowledge about breast cancer positively influences the screening behavior of women and encourages them to seek medical help at the time of detecting the initial signs and symptoms [12]. Evidence suggests limited knowledge and various sociodemographic factors contribute to delayed medical seeking behavior if initial signs and symptoms of breast cancer go undetected [13]. It is, therefore, essential to assess females’ knowledge of breast cancer and understand the factors that might prevent women from seeking early medical attention when encountering suspicious symptoms.

One of the Gulf Cooperation Council states, the state of Qatar offers free universal health care to its residents and citizens. Although several private clinics and hospitals operate in the country, approximately 70% of the population is registered with and receives primary care via the Primary Health Care Corporation (PHCC) [14]. Hamad Medical Corporation (HMC) delivers secondary and tertiary care and, as such, is the country’s primary site for cancer treatment [15]. In October of each year, under the umbrella of the Ministry of Public Health—National Cancer Program, these institutions promote awareness of breast cancer screening in public spaces in the country. In addition, the Qatar Cancer Society (QCS) takes part in promoting knowledge and awareness on breast cancer screening and interventions. The most recent awareness campaign occurred in 2020 and focused on breast cancer signs, symptoms, risk factors, prevention, and screening.

However, despite the coordinated efforts of these organizations, community knowledge of the National Breast Screening Program (NBCS) remains suboptimal. The majority of women who participated in the study indicated they neither know the starting age for invitation for the NBCS program (4090, 45.4%) nor the age women receive their last invitation (7036, 78%). Only 18% of respondents acknowledged that they received an invitation for breast screening (1600, 18%) and of those, 17% subsequently attended the NBCS (1510/1600, 94%). These figures suggest there is scope for improvement regarding public awareness of breast screening.

Over the past decade, an increasing number of studies have examined Arab women’s knowledge about the signs and symptoms of breast cancer and the risk factors of the disease [16,17,18,19,20]. A few of these studies were carried out in Qatar a decade ago, with a focus on Arab women’s breast cancer awareness and knowledge of local Breast Cancer Screening (BCS) programs [16,17,18]. However, Qatari research has excluded non-Arab residents and largely excluded women of all ethnicities under the age of 30. Thus, there is a significant gap in the data pertaining to Australian, Southeast Asian, European, and North and South American females who also access healthcare in Qatar. The aim of this study was to acquire current baseline data of women’s knowledge, attitudes, and practices of Breast self-examination (BSE) and BCS.

## 2. Materials and Methods

A list of mobile phone numbers for all women over 18 years of age and registered with a PHCC health center was extracted from the PHCC’s electronic medical record system. A Short Message Service (SMS) message in Arabic and English was sent to 440,350 women’s mobiles inviting them to participate in the study in March–April 2021. The SMS included a link to a questionnaire hosted on Microsoft Forms. The first page of the questionnaire included a participant information form and an informed consent sheet, which required a signature prior to participation in the survey. The questionnaire administered was a modified version of the Breast Cancer Awareness Module (Breast-CAM) (The Breast Module of the Cancer Awareness Measure (Breast-CAM)* was developed by Cancer Research UK, King’s College London, and University College London in 2009 and was validated with the support of Breast Cancer Care and Breakthrough Breast Cancer) developed by Cancer Research UK, King’s College London and University College London in 2009 and validated with the support of Breast Cancer Care and Breakthrough Breast Cancer. The tool was back-translated, reviewed by native Arabic and English speaking researchers, and piloted among a small cohort of patients before dissemination. The questionnaire remained open for completion for four weeks. A reminder SMS was sent after 2 weeks.

All data were collated and subject to quality assurance. The data were reviewed and cleaned before they were analyzed. Data were collected followed organizational safeguarding policies including encrypted storge and anonymous analysis. Descriptive statistics (frequency) and chi-squared tests were performed to determine any associations between categorical dependents and predictors. Statistical significance was established at alpha −0.05. Data analysis was conducted using SPSS version 25(IBM, Armonk, NY, USA).

The study was reviewed and approved by Primary Health Center Corporation’s Research Sub-Committee (PHCC/DCR/2020/09/108) and Qatar University (QU-IRB 1519-EA/21). Informed consent was obtained online. No participant identifiable information was collected. Only EH and MS had access to the study data. Overall, the study was conducted with integrity according to generally accepted ethical principles.

## 3. Results

The comprehensive online survey yielded a response rate of 2.04%. Of the 9008 women who participated in the survey, more than half were under 40 years of age (56%). Arabic was the predominant spoken language. The most common nationalities who participated were Qatari and North African, both at 20%. The majority of respondents (83%) were educated to the college level diploma or above. More than two-thirds of respondents purported an awareness of the warning signs of breast cancer 69% (Table 1).

When completing the survey, women could select more than one response. The most commonly recognized sign amongst the participants was a lump or thickening under the armpit (4893, 54%), followed by a lump in the breast or thickening of the breast tissue (4585, 50.9%). Other symptoms and warning signs were not recognized by more than half of the respondents. More than two-thirds of the respondents correctly identified “Past medical history of breast cancer” and “having a close relative with breast cancer” as risk factors for breast cancer. However, early menarche, late menopause, and advanced childbearing age were not readily recognized as risk factors by the survey cohort (Table 2).

Half of the respondents refrained from selecting a specific age group contending that women of any age were likely to get breast cancer (4179, 46.7%), nor was there any certainty expressed about the incidence rate of women who will develop breast cancer in their lifetime (3118, 35%).

Nearly half of the respondents admit to rarely or never checking their breasts for abnormalities (3979, 44.6%). Of those participants that do perform SBE: a fifth only check at least once every 6 months (1781, 19.95%); a similar number contend checking at least once a month (1645, 18.43%) and a minority indicated they do so at least once a week (740, 8.3%). While a minority felt very confident in noticing a change in the breast (804, 8.9%), nearly a fifth of the survey responses reported they were not confident (1806, 20%). Nearly a third of the respondents indicated that they saw a doctor about a change they have noticed in one of their breasts (2619, 29.25%), and more than half indicated that upon detection of an abnormality, they would contact their doctor as soon as possible (4711, 52.3%) (Table S2).

In general, women conveyed the sense that they found healthcare provision accessible and doctors approachable, harboring little fear of embarrassment (70%), concerns about wasting physician time (81%), or experiencing any difficulties communicating with their doctors (72%). The most common barrier to seeking medical help reported was difficulty making appointments with the doctor (60%). Half of the women cited sometimes or often being too busy to make time to go to the doctor (49%). Despite a majority claiming not being “too scared to go and see the doctor” and seeking medical help, just under half of the respondents (3562, 43%) admitted “being worried about what the doctor might find.” Thus, the specific fear of diagnosis was a significant deterrent to health-seeking behavior (Table 3).

## 4. Discussion

This study examined knowledge of symptoms and warning signs, awareness of risk factors of breast cancer, and barriers to seeking medical help amongst women in Qatar. The survey included women from all nationalities and sociocultural and sociodemographic backgrounds. To the best of our knowledge, no other study has used a validated measure to assess cancer knowledge, attitudes, and practices in Qatar. The results of BCS practices captured in this 2021 survey are consistent with earlier reported studies and results from other countries in the Gulf and Middle East [17,18,19,21,22,23].

While Qatari nationals represent only 10% of the total population, 20% of women (*n* = 1786) completing the survey identified as Qatari females; consequently, it is likely that a higher proportion of the survey population are Qatari than in the whole population of Qatar. The fact that Qataris were more likely to respond is also mirrored in their uptake of screening services.

Despite the availability of free health care services and a recent national awareness campaign in 2020 focusing on signs and symptoms, risk factors, prevention, and screening of breast cancer, the majority of women continue to have low awareness of signs and symptoms, risk factors, and the current screening program. This concurs with Donnelly et al.’s 2014 findings, which found women’s knowledge of national guidelines for breast screening practices in Qatar to be low [16]. This study reveals similar results. While almost 70% of respondents contended to know the warning signs of breast cancer, when asked about specific signs, these results were not substantiated. On average less than half of respondents recognized signs positive for breast cancer. Very few warning signs (3/11) were readily recognized as positive indicators; half of the women were able to correctly identify a lump or thickening under the armpit pain in breast or armpit and a lump or thickening in one’s breast. Similarities may be that these particular signs are more easily recognized having become mainstream knowledge as a result of films, television programs, and social media.

Of the positive risk factors of breast cancer, only two were readily identified: having a past history of breast cancer and having a close relative with breast cancer. The remaining six risk factors prompted the majority of women to declare that they were “not sure.” There appears to be much uncertainty about the link of using hormone replacement therapy and the consumption of alcohol. Qatar, being a Muslim-majority nation, might account for the lack of knowledge pertaining to the impact of drinking alcohol as this practice is prohibited in Islam.

Many studies correlate that higher education level is a strong predictor for BCS awareness, yet the findings of our highly educated cohort contravene this assertion [16,17]. Over 83% of the respondents were educated to the college level, yet their recall of the vast majority of warning signs was poor as was their participation in CBE. This suggests that other factors other than education must be at play in terms of poor awareness. Unlike an earlier study, this research did not evaluate the impact of the male family members’ levels of education on BSC knowledge and awareness [16,17]. Given the integral role males play in health-making decisions, educational campaigns should be targeted to both genders for maximum impact. Further, an assessment of resident males’ knowledge and attitudes surrounding breast screening practices requires consideration in this social context to determine if their awareness impacts their female counterpart’s participation in screening behaviors.

In Qatar, an earlier study reported rates of BSE at least once a month of 13.8% and BCS programs in Qatar as low as 4% [24]. Though still low, our results indicate that the number of women who practice BSE has nearly doubled at 25%, and the number of women who had attended the BCS program has quadrupled to 16%. This may indicate that targeted awareness campaigns have succeeded in educating this Arabic demographic. Notably, Qatari women and young females were among the lowest groups to practice BSE; thus, targeted campaigns should address these demographics to aid in earlier detection. Further work should focus on increasing the awareness among these groups, though they are less at risk. Though these figures have improved compared to the studies conducted in 2013 and 2015, it is difficult to measure to what extent clinical debates surrounding the efficacy of SBE may have adversely affected this practice in the intervening period. A similar study conducted in Saudi Arabia found “shyness” and “not wanting to be examined by a male physician” to be the main reasons that women did not seek CBEs [21]. Although Qatar has a similar gender-appropriate healthcare system and shares many sociocultural and religious parallels with Saudi Arabia, 70% contend they are not embarrassed and 61% claimed they were not too scared to visit the doctor. Though our survey did not explicitly inquire if respondents preferred male or female physicians, almost three quarters (72%) state that they find their physicians approachable and “not difficult to talk to”.

Barriers to attending the BCS program differed from earlier work reflecting the change of demographics. In our population, time, availability, and accessibility are reported as significant barriers compared to health beliefs. Earlier studies in the Qatari society reported health beliefs and fear of the findings as more common [17,18,24]. Understandably, fear still continues to resonate strongly among approximately one third of the study population.

Region was the factor that had the most significant impact on breast self-examination. Women from Qatar, Africa, and Asia practice BSE once a month or less frequently compared to women from the Americas, Europe, and Australia. This result may highlight areas for improvement with a need for campaigns for breast cancer awareness and BSE in those groups who do not engage is BSE.

Donnelly et al.’s study of female Arabic-speaking residents of Qatar concluded that nationality did not affect participation rates in BCS activities, attributing this to the fact that screening services are free or heavily subsidized [24]. Our study indicates that women from Qatar and Northern Africa have the highest uptake of BCS in the country. This may reflect that these groups are more aware of the local BCS local program, perhaps a direct consequence of Arabic-medium awareness campaigns targeting the Arab community. Indeed, groups from Europe, the Americas, and Australia may not be attending the program in Qatar as they are engaged with the program in their own local countries, preferring continuity of care in their home countries.

Though figures are unavailable, it is also possible that women are engaging in BCS in private clinics or abroad, neither of which would be captured in the survey disseminated by the PHCC, so the uptake of screening may be higher than reported here. These clinics may be perceived to afford more privacy and circumvent any stigma associated with breast cancer diagnosis that might occur in a small, closely-related community. Prior to the establishment of national screening programs and cancer treatment being readily available in Qatar, citizens were funded to travel abroad for diagnosis and treatment. A legacy of this practice may be that it has undermined confidence in local healthcare provision. Advertising the high caliber of expertise and advanced screening technology available in Qatar as well as celebrating local advances in breast cancer prevention and care could mitigate some of these issues.

The Health Belief Model holds that a patient’s willingness to change health behaviors is contingent on their perceived susceptibility and severity as well as the perceived benefits and barriers associated with a particular health-seeking behavior. Uptake of screening occurs when a person feels the risk of disease is significant for them. It is likely, for instance, that the perception of low susceptibility to breast cancer accounts for why women under 39 years of age rarely or never perform SBE. Further education is needed to alert females to the signs and symptoms, and risk factors, in developing breast cancer so they can make informed decisions about engaging with breast screening from an early age. Amin et al. suggest that Arab patients tend to visit physicians only upon the onset of illness or the appearance of symptoms [21]. This observation has an important implication; if women conflate screening with treatment, they may not appreciate the need to go for regular mammograms if they are asymptomatic. Campaigns conveying clear messages about cancer prevention, the effectiveness of BCS in early detection, as well as survival rates and advances in cancer care would be beneficial.

Daher-Nashif and Bawadi found that in the GCC particularly, women’s health in general is shaped by the sociocultural factors that also shape health policies and institutional practices [25]. Reporting on the psychosocial aspects of female breast cancer in the Middle East and Africa, Salem and Daher-Nashif argue that sociocultural factors such as religion and cultural values have a marked influence on BSE and BCS behaviors. They contend that there is a fissure between their beliefs that diagnosis of cancer can be perceived as “a punishment or test that only God can heal” and the belief that “one’s body is a gift from God and that it is ones’ responsibility to care for it” [26]. This contradiction is reflected in some Muslim women’s screening behavior in Qatar whereby those with fatalistic views may be less inclined to screen, ascribing a cancer diagnosis to God’s will, whereas the women that engage in BCS may regard it as a proactive means of caring for their body. Further qualitative research exploring the motivations amongst this latter cohort could reveal important insights that might be capitalized on in the design of future campaigns.

### 4.1. Clinical Implications

These baseline data demonstrate a persistent lack of awareness about BSE and an underutilization of screening services in Qatar despite recent breast cancer campaigns. Information communicated by healthcare providers is a strong predictor of BCS awareness [16]. Though beyond the scope of the Breast-CAM tool employed in this study, further research is required to identify what sources of information women in Qatar rely on for accurate information pertaining to breast health and to what extent healthcare professionals actively refer women for BCS as part of their routine visits. Of interest, most women who received an invitation for BCS had attended, notwithstanding time and fear factors. These encouraging findings indicate that the screening program uptake could be improved dramatically with appropriate interventions geared to increase physician breast cancer knowledge and referrals.

The high rate of responses indicate that the modified Breast-CAM is a culturally acceptable tool among the different Arabic and English-speaking ethnic groups in Qatar and is a useful mechanism for generating a comprehensive assessment of breast cancer awareness in a Middle Eastern context. Used regularly, this tool could provide a useful measure of changes in attitude and behaviors surrounding breast health.

### 4.2. Study Limitations

This survey was administered only in Arabic and English; consequently, results may not represent the minority population who are non-Arabic or non-English speakers or are illiterate. Similarly, individuals who prefer private healthcare and do not register with the PHCC will not be represented in this survey. Another factor that may influence the responses obtained is the familiarity expatriate females have with the Qatar health care system. The survey did not identify the number of years spent in Qatar at the time of completion of the survey, so consequently, a limitation of the interpretation arises since the information the participants have may be influenced by the strength of awareness of breast cancer campaigns from their country of origin. This is particularly relevant for new arrivals to Qatar.

## 5. Conclusions

The current study findings imply an improvement in the knowledge and practices of BCS in Qatar, although there is significant room for improvement. There is a disconnect between participants’ perceived awareness of their ability to detect breast cancer and their actual recognition of individual signs and symptoms of breast cancer. Similarly, their BSE and BCS uptake is low. BCS invitation is remarkably effective in improving the uptake. Policymakers should consider the employment of the intervention for the national program and capitalize on as early recognition as possible in the youthful population. Awareness campaigns and interventions targeting a broader audience including males, community and religious leaders, and healthcare professionals may prove more effective in Arab communities. Further qualitative research should be undertaken to determine what factors are inhibiting the acquisition and subsequent application of SBE and CBE in these highly educated women.

## Figures and Tables

**Table 1:** Participant characteristics.

Participant Characteristics	Response (*n* = 9008)	
	N	%
**Age (years)**		
18–29	1724	19
30–39	3394	37
40–49	2482	28
50–59	1191	13
60 and above	224	2.5
Not reported	38	0.4
**Nationality**		
Qatari	1786	20
North African	1803	20
Sub-Saharan African	177	2
Northern American	146	1.6
South American and Latin American	80	0.9
South-eastern Asian	1.094	12
Southern Asian (excluding Qatar)	1162	13
Western Asian	1736	19
European	487	5.4
Not reported	537	6
**Legnth of stay in Qatar**		
Less than 5 years	2038	23
5–10 years	3492	39
10–15 years	2077	23
More than 15 years	3531	39
No answer	1799	20
**Main spoken language**		
Arabic	5633	63
English	2846	32
Both	30	0.3
Others	499	5.5
**Highest level of education**		
Never attended school	1	<0.1
Primary school (Class 1 to 6)	61	1
Secondary school (Class 7 to 12)	1109	12
Trade/technical/vocational qualification	227	3
College diploma/certificate	1293	14
University bachelor’s degree	4712	52
Postgraduate degree	1570	17
Not reported	31	0.34
**Average monthly household income (QR)**		
Up to 4999	1441	17
5000–9999	1897	22
10,000–14,999	1506	18
15,000–19,999	900	11
>20,000	2766	33
Not reported	498	
**Do you know of the warning signs of breast cancer?**		
Yes	6251	69

**Table 2:** Awareness of the warning signs and risk factors of breast cancer.

Awareness of the Warning Signs of Breast Cancer	Overall (N = 9008)
Change in position of your nipple	2489 (27.6%)
Pulling in of your nipple	2816 (31.3%)
Pain in one of your breasts or armpits	4654 (51.7%)
Puckering or dimpling of your breast skin	3516 (39.0%)
Discharge or bleeding from your nipple	4295 (47.7%)
A lump or thickening in your breast	4585 (50.9%)
Nipple rash	1717 (19.1%)
Redness of your breast skin	2383 (26.5%)
A lump or thickening under your armpit	4893 (54.3%)
Changes in the shape of your breast or nipple	4044 (44.9%
Changes in the size of your breast or nipple	3687 (40.9%)
**Awareness of the risk factors of breast cancer**	
**How much do you agree that each of these can increase the chance of getting breast cancer?**	
**Having a past history of breast cancer**	N-Miss 368
Strongly Agree	2337 (27.1%)
Agree	3785 (43.8%)
Not Sure	1427 (16.5%)
Disagree	355 (4.1%)
Strongly Disagree	736 (8.5%)
**Using HRT (Hormone Replacement Therapy)**	N-Miss 500
Strongly Agree	738 (8.7%)
Agree	2951 (34.7%)
Not Sure	3927 (46.2%)
Disagree	457 (5.4%)
Strongly Disagree	435 (5.1%)
**Drinking more than 1 unit of alcohol a day**	N-Miss 547
Strongly Agree	735 (8.7%)
Agree	1987 (23.5%)
Not Sure	4188 (49.5%)
Disagree	954 (11.3%)
Strongly Disagree	597 (7.1%)
**Being overweight (BMI over 25)**	N-Miss 512
Strongly Agree	497 (5.8%)
Agree	2231 (26.3%)
Not Sure	4171 (49.1%)
Disagree	1153 (13.6%)
Strongly Disagree	444 (5.2%)
**Having a close relative with breast cancer**	N-Miss 425
Strongly Agree	2036 (23.7%)
Agree	3845 (44.8%)
Not Sure	1442 (16.8%)
Disagree	543 (6.3%)
Strongly Disagree	717 (8.4%)
**Having children later on in life (after 30 years old) or not at all**	N-Miss 564
Strongly Agree	310 (3.7%)
Agree	1290 (15.3%)
Not Sure	4323 (51.2%)
Disagree	1717 (20.3%)
Strongly Disagree	804 (9.5%)
**Starting your periods at an early age (before 12 years old)**	N-Miss 578
Strongly Agree	246 (2.9%)
Agree	855 (10.1%)
Not Sure	4550 (54.0%)
Disagree	1924 (22.8%)
Strongly Disagree	855 (10.1%)
**Having a late menopause (after 55 years old)**	N-Miss 580
Strongly Agree	205 (2.4%)
Agree	929 (11.0%)
Not Sure	4900 (58.1%)
Disagree	1650 (19.6%)
Strongly Disagree	744 (8.8%)
**Doing less than 30 min of moderate physical activity 5 times a week**	N-Miss 549
Strongly Agree	393 (4.6%)
Agree	1692 (20.0%)
Not Sure	4009 (47.4%)
Disagree	1492 (17.6%)
Strongly Disagree	873 (10.3%)

**Table 3:** Barriers to seeking medical help.

Characteristic	N = 9008
**Too embarrassed to go and see the doctor**	
I don’t know	144 (1.7%)
No	5841 (70%)
Yes often	671 (8.0%)
Yes sometimes	1691 (20%)
Unknown	661
**Too scared to go and see the doctor**	
I don’t know	104 (1.2%)
No	5078 (61%)
Yes often	1081 (13%)
Yes sometimes	2117 (25%)
Unknown	628
**Worried about wasting the doctor’s time**	
I don’t know	205 (2.5%)
No	6702 (81%)
Yes often	358 (4.3%)
Yes sometimes	1009 (12%)
Unknown	734
**I find my doctor difficult to talk to**	
I don’t know	212 (2.6%)
No	5979 (72%)
Yes often	480 (5.8%)
Yes sometimes	1597 (19%)
Unknown	740
**Difficult to make an appointment with the doctor**	
I don’t know	162 (1.9%)
No	3232 (38%)
Yes often	2412 (28%)
Yes sometimes	2678 (32%)
Unknown	524
**Too busy to make time to go to the doctor**	
I don’t know	152 (1.8%)
No	4083 (49%)
Yes often	1208 (14%)
Yes sometimes	2926 (35%)
Unknown	639
**Too many other things to worry about**	
I don’t know	361 (4.3%)
No	4394 (53%)
Yes often	1135 (14%)
Yes sometimes	2423 (29%)
Unknown	695
**Difficult to arrange transport to the doctor’s surgery**	
I don’t know	303 (3.7%)
No	6593 (80%)
Yes often	379 (4.6%)
Yes sometimes	974 (12%)
Unknown	759
**Worrying about what the doctor might find may stop me from going to the doctor**	
I don’t know	201 (2.4%)
No	4595 (55%)
Yes often	1412 (17%)
Yes sometimes	2151 (26%)
Unknown	649
**Not feeling confident talking about my symptom with the doctor**	
I don’t know	223 (2.7%)
No	6207 (75%)
Yes often	535 (6.5%)
Yes sometimes	1317 (16%)
Unknown	726
**My doctor does not understand my language or culture**	
I don’t know	293 (3.5%)
No	5942 (72%)
Yes often	594 (7.2%)
Yes sometimes	1476 (18%)
Unknown	703
**Statistics presented: *n* (%)**	

Younger age groups (18–40 years) responded with less awareness and reported practicing BSE and BCS less often than older women (Table S1). Qatari females were among the highest groups who do not practice BSE (982, 56%) though their rates for attending BCS were the highest (501, 28%) (Table S2).

## Data Availability

The data presented in this study are available on request from the corresponding author. The data are not publicly available due to privacy issues.

## Supplementary Materials

The following supporting information can be downloaded at: https://www.mdpi.com/article/10.3390/ijerph19073995/s1, Table S1: Age as predictor for awareness and practices of BCS among the population of Qatar. Table S2: Region as a predictor of awareness and practices of BCS.
