# Supplementary material for: Breast Cancer Knowledge, Attitudes and Practices amongst Women in Qatar
Source: Int J Environ Res Public Health. 2022 Mar 28;19(7):3995. doi: 10.3390/ijerph19073995 (PMC8997898; doi:10.3390/ijerph19073995)
Supplement: Supplementary file 1 [file ijerph-19-03995-s001.zip › ijerph-1589821-supplementary.pdf]

Supplemental Table S1: Age as predictor for awareness and practices of BCS among the population of Qatar

**Characteristic**	**N**	**18-29**<br>**N = 1698 (19%)**	**30-39**<br>**N = 3313 (37%)**	**40-49**<br>**N = 2477 (28%)**	**50-59**<br>**N= 1179**<br>**(13%)**	**>60**<br>**N = 223 (2.5%)**
**How often do you check your breasts? Please tick one box only.**	8,890					
Rarely or never		932 (55%)	1,578 (48%)	1,002 (40%)	396 (34%)	52 (23%)
At least once every 6 months		243 (14%)	618 (19%)	542 (22%)	295 (25%)	77 (35%)
At least once a month		193 (11%)	560 (17%)	529 (21%)	302 (26%)	53 (24%)
At least once a week		139 (8.2%)	241 (7.3%)	219 (8.8%)	118 (10%)	22 (9.9%)
Don't know		191 (11%)	316 (9.5%)	185 (7.5%)	68 (5.8%)	19 (8.5%)
**Have you ever had breast screening on the Qatar Breast Screening Program?**	8,892					
Don't know		26 (1.5%)	25 (0.8%)	27 (1.1%)	10 (0.8%)	2 (0.9%)
No		1,621 (96%)	3,188 (96%)	1,972 (80%)	451 (38%)	62 (28%)
Yes		46 (2.7%)	104 (3.1%)	473 (19%)	726 (61%)	159 (71%)

Pearson's Chi-squared test

Supplemental Table S2: Region as a predictor of awareness and practices of BCS

Characteristic	Qatar N = 1786 (20%)	Western Asia N = 1736 (20%)	Southern Asia N = 1162 (13%)	South-eastern Asia N = 1094 (12%)	Eastern-Central Asia N = 26 (0.3%)	Northern Africa N = 1803 (20%)	Sub-Saharan Africa N = 177 (2.0%)	Northern America N = 146 (1.7%)	South America N = 80 (0.9%)	Australasia N = 37 (0.4%)	Europe N = 487 (5.5%)	Other N = 309 (3.5%)
**How often do you check your breasts? Please tick one box only. N = 8,769**												
**Rarely or never**	982 (56%)	833 (49%)	452 (39%)	379 (35%)	12 (46%)	848 (47%)	65 (37%)	44 (31%)	19 (24%)	10 (27%)	115 (24%)	141 (46%)
**At least once every 6 months**	322 (18%)	376 (22%)	207 (18%)	193 (18%)	9 (35%)	316 (18%)	36 (20%)	47 (33%)	29 (36%)	12 (32%)	147 (30%)	59 (19%)
**At least once a month**	176 (10.0%)	242 (14%)	245 (21%)	347 (32%)	4 (15%)	317 (18%)	43 (24%)	35 (24%)	21 (26%)	11 (30%)	138 (29%)	44 (14%)
**At least once a week**	104 (5.9%)	117 (6.8%)	143 (12%)	118 (11%)	1 (3.8%)	109 (6.1%)	23 (13%)	13 (9.1%)	8 (10%)	2 (5.4%)	64 (13%)	28 (9.1%)
**Don’t know**	179 (10%)	144 (8.4%)	113 (9.7%)	56 (5.1%)	0 (0%)	197 (11%)	10 (5.6%)	4 (2.8%)	3 (3.8%)	2 (5.4%)	20 (4.1%)	35 (11%)
**Have you ever had breast screening on the Qatar**												

**Breast Screening Program?**<br><br>**N = 8,782**												
**Don't Know**	27 (1.5%)	15 (0.9%)	15 (1.3%)	8 (0.7%)	0 (0%)	16 (0.9%)	0 (0%)	3 (2.1%)	0 (0%)	0 (0%)	4 (0.8%)	2 (0.6%)
**No**	1,252 (70%)	1,426 (83%)	1,004 (88%)	958 (88%)	23 (88%)	1,558 (87%)	149 (85%)	122 (84%)	69 (86%)	29 (78%)	379 (78%)	252 (82%)
**Yes**	501 (28%)	280 (16%)	126 (11%)	124 (11%)	3 (12%)	212 (12%)	26 (15%)	21 (14%)	11 (14%)	8 (22%)	104 (21%)	55 (18%)
